# Support Induced Effects on the Ir Nanoparticles Activity, Selectivity and Stability Performance under CO_2_ Reforming of Methane

**DOI:** 10.3390/nano11112880

**Published:** 2021-10-28

**Authors:** Ersi Nikolaraki, Grammatiki Goula, Paraskevi Panagiotopoulou, Martin J. Taylor, Kalliopi Kousi, Georgios Kyriakou, Dimitris I. Kondarides, Richard M. Lambert, Ioannis V. Yentekakis

**Affiliations:** 1 Laboratory of Physical Chemistry and Chemical Processes, School of Chemical and Environmental Engineering, Technical University of Crete, 73100 Chania, Crete, Greece; enikolaraki@isc.tuc.gr (E.N.); matgoula@gmail.com (G.G.); ppanagiotopoulou@isc.tuc.gr (P.P.); 2 Energy and Environment Institute, University of Hull, Hull HU6 7RX, UK; Martin.Taylor@hull.ac.uk; 3 Department of Chemical and Process Engineering, University of Surrey, Guildford GU2 7XH, UK; k.kousi@surrey.ac.uk; 4 Department of Chemical Engineering, University of Patras, GR 265 04 Patras, Greece; kyriakg@upatras.gr (G.K.); dimi@chemeng.upatras.gr (D.I.K.); 5 Energy & Bioproducts Research Institute (EBRI), Aston University, Aston Triangle, Birmingham B4 7ET, UK; 6 Department of Chemistry, Cambridge University, Cambridge CB2 1EW, UK; rml1@cam.ac.uk; 7 Institute of Petroleum Research—Foundation for Research and Technology-Hellas (IPR-FORTH), 73100 Chania, Crete, Greece

**Keywords:** greenhouse gases, dry reforming of methane, carbon dioxide, alumina-ceria-zirconia mixed oxides, iridium nanoparticles, coking-resistant catalysts, sintering-resistant catalysts, syngas production

## Abstract

The production of syngas (H_2_ and CO)—a key building block for the manufacture of liquid energy carriers, ammonia and hydrogen—through the dry (CO_2_−) reforming of methane (DRM) continues to gain attention in heterogeneous catalysis, renewable energy technologies and sustainable economy. Here we report on the effects of the metal oxide support (γ-Al_2_O_3_, alumina-ceria-zirconia (ACZ) and ceria-zirconia (CZ)) on the low-temperature (ca. 500–750 °C) DRM activity, selectivity, resistance against carbon deposition and iridium nanoparticles sintering under oxidative thermal aging. A variety of characterization techniques were implemented to provide insight into the factors that determine iridium intrinsic DRM kinetics and stability, including metal-support interactions and physicochemical properties of materials. All Ir/γ-Al_2_O_3_, Ir/ACZ and Ir/CZ catalysts have stable DRM performance with time-on-stream, although supports with high oxygen storage capacity (ACZ and CZ) promoted CO_2_ conversion, yielding CO-enriched syngas. CZ-based supports endow Ir exceptional anti-sintering characteristics. The amount of carbon deposition was small in all catalysts, however decreasing as Ir/γ-Al_2_O_3_ > Ir/ACZ > Ir/CZ. The experimental findings are consistent with a bifunctional reaction mechanism involving participation of oxygen vacancies on the support’s surface in CO_2_ activation and carbon removal, and overall suggest that CZ-supported Ir nanoparticles are promising catalysts for low-temperature dry reforming of methane (LT-DRM).

## 1. Introduction

The reforming of methane by carbon dioxide for the production of synthesis gas (H_2_ + CO), the so-called dry reforming of methane (DRM; reaction R.1), ranks among the top issues of heterogeneous applied catalysis in the light of renewable energy production, environmental protection, and sustainable economy [1,2,3,4]. DRM concerns the simultaneous utilization of CO_2_ and CH_4_ (two key greenhouse gases) and provides an effective method for the recycling of carbon dioxide via a more sustainable approach to natural gas utilization, as well as for the direct use of biogas [3,4,5,6,7,8,9]. Furthermore, in light of the DRM energy applications, the produced syngas is a very suitable fuel for solid oxide fuel cells (SOFCs) operating at intermediate and high temperatures, or even, after CO removal, for low-temperature polymer electrolyte membrane fuel cells (PEM-FCs), toward electrical power generation [3,4]. Το this end, an enhanced electrical power efficiency and energy saving concept is the direct biogas-fueled solid oxide fuel cells (DB-SOFCs). This process, also called *internal dry reforming of methane* (In-DRM), involves the simultaneous occurrence of the catalytic DRM reaction (R.1) with the H_2_ + O^2−^ → H_2_O + 2e−, CO + O^2−^ → CO_2_ + 2e− and C + O^2−^ → CO_2_ + 2e− charge transfer reactions that produce electricity, all together operated onto the anodic electrode of the SOFC [3,10,11,12,13,14]. The advantages of this system are: (i) process simplicity and economy of installation and operating cost, with no need for an external reformer, and (ii) minimization of energy losses due to an improved in situ heat exchange between the charge transfer reactions (exothermic) and the DRM reaction (endothermic). The combination of the coordinated operation of municipal and agricultural wastewater treatment plants that produce biogas with an In-DRM fuel cell system converting the biogas chemical energy to electricity can be beneficial from both the environmental and energy points of view, since it can lead to the establishment of a large number of decentralized electricity production units [3,4,11]. Currently, the dry reforming of methane process as well as the hydrogenation of CO_2_ to methane (the so-called Sabatier reaction [15,16,17]) are considered as the most promising alternatives for CO_2_ emission control via its sustainable recycling [1,4].

The above advantages suggest that the DRM process is more favorable compared to alternative concepts, such as steam reforming (SRM; R.2) and oxy reforming of methane (ORM; R.3):

*DRM*:   CH_4_ + CO_2_ ⇋ 2CO + 2H_2_     ΔH^ο^_298_ = +247 kJ mol^−1^    (R.1)

*SRM*:  CH_4_ + H_2_O ⇋ CO + 3H_2_    ΔH^ο^_298_ = +206 kJ mol^−1^    (R.2)

*ORM*:  CH_4_ + ½ O_2_ ⇋ CO + 2H_2_    ΔH^ο^_298_ = −36 kJ mol^−1^    (R.3)

Over the temperature range 650–800 °C, where DRM is typically operated, other side reactions are favored too. These mainly include the reverse water-gas shift reaction (rWGS; R.4), methane cracking (MC; R.5), the Boudouard reaction (BR; R.6), carbon gasification (CGR; R.7) and deep or partial carbon oxidation reactions (DCO or PCO; R.8 and R.9, respectively) [1,2,3,4,5,6,7,8,9]:

*rWGS:*  H_2_ + CO_2_ ⇄ CO + H_2_O      ΔH^ο^_298_ = +41 kJ mol^−1^    (R.4)

*MC*:  CH_4_ ⇄ 2H_2_ + C      ΔH^ο^_298_ = +75 kJ mol^−1^    (R.5)

*BR*:  2CO ⇄ CO_2_ + C       ΔH^ο^_298_ = −172 kJ mol^−1^    (R.6)

*CGR*:  C + H_2_O ⇄ CO + H_2_       ΔH^ο^_298_ = +131 kJ mol^−1^    (R.7)

*DCO*:  C + O_2_ → CO_2_      ΔH^ο^_298_ = −394 kJ mol^−1^    (R.8)

*PCO*:   C + ½ O_2_ → CO      ΔH^ο^_298_ = −110 kJ mol^−1^    (R.9)

Reactions (R.4)–(R.9) affect the H_2_/CO ratio of the produced syngas and the cumulative deposition of carbon, which is a major drawback in various DRM catalytic processes that have been investigated so far, as it determines catalyst stability with time-on-stream (TOS) [1,7,8,18,19,20,21,22,23]. Accordingly, the development of active and stable DRM catalysts, especially for use at low temperatures, remains a major research challenge in the field [1,5,24,25].

Due to their low cost and substantial DRM activity, Ni catalysts have been widely studied for DRM applications [7,8,20,21,22,23]. However, most these studies show that Ni-based catalysts lose their activity due to substantial carbon deposition and severe thermal sintering. The use of nano-dispersed Ni particles [26] or supports with enhanced basicity and/or high lattice oxygen ion lability and concentration of oxygen ion vacancies, including, among others, single or mixed oxides such as CeO_2_, ZrO_2_, TiO_2_, SiO_2_, SBA-15, MCM-41, La_2_O_3_, Nb_2_O_5_, MgO, CaO, Sm_2_O_3_, Pr_2_O_3_, Yb_2_O_3_, Y_2_O_3_, MgO-Al_2_O_3_, MgO-CaO-Al_2_O_3_, ZrO_2_-Al_2_O_3_, CeO_2_-Al_2_O_3_, La_2_O_3_-Al_2_O_3_, ZrO_2_-SiO_2_, Gd_2_O_3_-CeO_2_, CeO_2_-ZrO_2_, Al_2_O_3_-CeO_2_-ZrO_2_, WO_3_-ZrO_2_ and CeO_2_-WO_3_-ZrO_2_, e.g., refs. [27,28,29,30,31,32,33,34,35,36,37,38,39,40,41,42,43,44], as well as bimetallic catalyst formulations [1,45], are strategies that have been used to minimize the aforementioned adverse effects in Ni DRM catalysis. Such active supports or bi-metallic combinations in DRM catalyst formulations were found to induce strong electronic metal-support or metal-to-metal interactions, as well as bifunctional reaction pathways, which play an advantageous role not only on the anti-coking resistance and anti-sintering performance of the catalysts but also on their activity and selectivity performance at the favored low-temperature region [1,6,27].

On the other hand, noble metal (NM) based catalysts display an enhanced DRM activity compared to Ni-based catalysts and are characterized by greater resistance to carbon deposition as well as better anti-sintering performance [1,2,46,47,48,49,50]. These advantages offset their high price for prospective large-scale application, especially when low noble metal loading (<ca. 1 wt%) with high dispersion NM-catalysts are designed and used [6,50]. Among the noble metals, Rh and Pt have been mostly investigated so far under DRM conditions [51,52,53,54,55,56,57,58,59,60,61], while few studies have been reported for Ru [62,63,64,65] and, especially, Ir [50]. A similar trend is apparent for studies concerning bimetallic NM-Ni based catalysts [1].

Regarding the sintering behavior of dispersed metal nanoparticles, literature results show that the stability of some common catalysts in their *metallic* state (reducing environments) generally decreases in line with their Hütting (T_H_ = 0.3 T_melting_) and Tamman (T_T_ = 0.5 T_melting_) temperatures, as for example Ru > Ir > Rh > Pt > Pd > Ni > Cu > Ag [66]. Typically, the T_H_ and T_T_ values provide a good indication of the temperatures at which surface and bulk atoms are mobilized, thus leading to agglomeration [66,67], although the phenomenon is also influenced by the metal–support adhesion energy and possible strong interactions that can drastically alter such simplified predictions. The T_H_ and T_T_ criteria for the prediction of the propensity of nanoparticles agglomeration are of much less use under oxidizing environments. In this case the phenomenon also depends on the volatility, thermal stability (some metal oxides decompose before T_H_ or T_T_ being reached) and the strength of the metal–oxide–support interaction [66,67,68]. Regarding noble metal nanoparticles stability, Fiedorow et al. [69] investigated the sintering of Pt, Rh, Ru, and Ir particles dispersed on a relatively inert support (γ-Al_2_O_3_) and obtained the Ir~Ru > Rh > Pt sinter-resistant sequence in a reducing environment, in agreement with the metals’ T_T_ values. However, under oxidizing atmospheres, they found that the stability sequence was Rh_2_O_3_ > PtO_2_ > IrO_2_ > RuO_2_, not matching the T_T_ order of the oxides (RuO_2_ (735K) > Rh_2_O_3_ (687K) > IrO_2_ (685K) > PtO_2_ (362K).

Efforts aiming at designing sinter-resistant catalysts have often employed strategies that enhance the interaction between the nanoparticle and the support [70]. Atom trapping, i.e., immobilization of isolated single atoms on support sites of materials providing surface lattice oxygen defects (that can act as trapping centers), is a novel, highly promising approach for developing sinter-resistant catalysts [68,70,71,72,73]. CeO_2_− or perovskite-based supports are examples of materials that are characterized by a substantial population of surface and bulk oxygen vacancies, therefore offering so-called labile lattice oxygen species that enable bonding with single metal atoms, and have recently been successfully implemented for this purpose [50,74,75,76]. This creates a renewed interest in using lower-cost noble metals Ir or Ru, which are effective in high-temperature applications such as DRM, without concerns about their stability. It is also worth noting that, depending on the specific properties of the metal and the support in combination with the thermal treating conditions used, the aforementioned methodology can result in the redispersion of the metal particles [68]. Therefore, it can be considered as a possible strategy for in situ catalyst particles redispersion through on-stream oxidative treatment of the catalysts at high temperatures.

Recently, we have comparatively studied the DRM performance and stability of Ir nanoparticles supported on γ-Al_2_O_3_, YSZ and GDC supports [50]. The study was mainly focused on integral (high conversion) conditions and the effects of the supports on both the time-on-stream stability of the catalysts and their robustness after exposure to intense thermal aging conditions in an oxidative environment. Due to the integral conditions used, catalytic performance results were unsuitable for discriminating possible support-induced effects on the intrinsic DRM activity and selectivity of Ir nanoparticles via metal-support interactions. On the other hand, it was demonstrated that Ir catalysts have a very stable time-on-stream performance independent of the supporting material used, while the effect of thermal aging under oxidative environments on their robustness was more complicated: GDC support with high oxygen storage capacity and lability effectively protected Ir nanoparticles against agglomeration in contrast to γ-Al_2_O_3_ and YSZ with low or moderate oxygen storage capacity, respectively, where Ir nanoparticles were found to be prone to extended agglomeration. These changes in particle size/morphology were accompanied by proportional effects on DRM performance in catalytic experiments conducted after samples aging.

In order to further study the Ir-catalyzed DRM reaction, in the present work, we focused on investigating low-temperature DRM performance at differential reaction conditions (i.e., kinetic regime) so as to decipher the role of metal-support interactions on the Ir intrinsic activity and selectivity by using supports with different values of oxygen storage capacity, namely γ-Al_2_O_3_, ACZ (80 wt% γ-Al_2_O_3_ – 20 wt% Ce_0.5_Zr_0.5_O_2−__δ_) and CZ (Ce_0.5_Zr_0.5_O_2−__δ_). The corresponding Ir/γ-Al_2_O_3_, Ir/ACZ and Ir/CZ catalysts were well-characterized by a variety of methods in order to reveal structure-activity correlations and metal-support interactions. Moreover, in order to elucidate and further generalize the validity of the conclusion made in ref. [50] that supports with high oxygen storage capacity/lability can stabilize metal particle nanostructures with concomitant benefits on catalysts’ DRM performance characteristics, two thermal aging protocols were applied to all catalysts followed by comparative tests of their DRM performance. It is demonstrated that Ir dispersed on supports with high OSC values (i.e., Ir/ACZ and Ir/CZ catalysts) exhibited higher CO_2_ consumption activity with lower apparent activation energies and improved selectivity towards syngas enriched in CO and lower coking. Results are explained by considering the ease of formation of O^2−^ vacancies which act as centers for dissociative adsorption of CO_2_ and a bifunctional reaction mechanism involving both metal and oxide phases. The same catalysts offered significantly better robustness than that of Ir/γ-Al_2_O_3_ after thermal aging under oxidative conditions.

## 2. Materials and Methods

### 2.1. Materials and Treatment Protocols

- *Supporting materials:* γ-Al_2_O_3_ in the form of cylindrical pellets were obtained from Engelhard (Engelhard de Meern B.V., The Netherlands) and ground into powder (ca. < 250 mesh). ACZ (80 wt% γ-Al_2_O_3_ – 20 wt% Ce_0.5_Zr_0.5_O_2−δ_) and CZ (Ce_0.5_Zr_0.5_O_2−δ_) supports were prepared by co-precipitation of the corresponding metal nitrate precursors, namely Al(NO_3_)_3_·9H_2_O, Zr(NO_3_)_2_·H_2_O and Ce(NO_3_)_3_·6H_2_O (99.5%; Alfa Aesar, Haverhill, MA, USA), followed by calcination for 1 h at 800 °C; details of the method can be found elsewhere [77].
- *Supported Ir catalysts:* The wet impregnation method was followed to prepare the low iridium loading (0.4–1.0 wt%, Table 1) catalysts supported on γ-Al_2_O_3_, ACZ and CZ as follows: powders of γ-Al_2_O_3,_ ACZ, or CZ supports were impregnated under continuous stirring at 75 °C in an aqueous solution of IrCl_3_·H_2_O (Abcr GmbH & Co KG, Karlsruhe, Germany) of appropriate concentration to lead to a nominal Ir loading of 1 wt%. After water evaporation, these suspensions were dried at 110 °C for 12 h in air, then heated at 400 °C for 2 h under 50% H_2_/He flow (50 mL/min) followed by heating at 850 °C for 1 h under 1% H_2_/He flow (50 mL/min) in order to reduce the metal and yield the corresponding catalysts, hereafter referred to as “fresh”. It is worth noting that application of a reduction step directly after catalyst suspension drying was beneficial to the formation of small metallic Ir nanoparticles on the support surfaces [50,74,75], in contrast to methods which, instead of a reduction step at this stage of the preparation, use an oxidation one that results to the formation of larger Ir particles [78].

After the evaluation of the catalytic performance of the samples in their “fresh” stage, these were subjected to the same aging protocols in order to establish their relative susceptibilities toward sintering. The aging protocols were as follows: (i) a first step of 2 h oxidation in 50 mL/min of 20% O_2_/He flow at 650 °C (such treated catalysts are denoted hereafter by the end term “aged@650”), and (ii) a consecutive step of oxidation for 2 additional hours in 50 mL/min of 20% O_2_/He flow at 750 °C (denoted hereafter by “aged@750”). After each step, the DRM performance of the samples at constant temperature (750 °C) and feed conditions (CH_4_:CO_2_ = 50%:50% with F_t_ = 100 mL/min at a total pressure of 1 bar) was re-evaluated.

### 2.2. Materials Characterization

The morphological, structural and textural characteristics of the samples were evaluated with a variety of techniques, including N_2_ adsorption-desorption (BET-BJH method), isothermal hydrogen chemisorption (H_2_-Chem.), powder X-ray diffraction (PXRD), inductively coupled plasma optical emission spectroscopy (ICP-OES) and high-resolution transmission electron microscopy (HR-TEM). The reducibility characteristics and total oxygen storage capacities of the samples were determined via hydrogen temperature-programmed reduction (H_2_-TPR) experiments. Temperature-programmed oxidation (TPO) measurements were used to determine the amount and kind of carbon deposited after 3 h in DRM operation. Specifically:

- *Textural characteristics:* A Quantachrome Nova 2200e instrument (Boynton Beach, FL, USA) was used to determine the BET surface areas of the samples through N_2_ adsorption–desorption isotherms at relative pressures in the range of 0.05–0.30 and a temperature of −196 °C. The samples were previously vacuum degassed at 350 °C for 12 h. The total pore volumes and pore size distributions were calculated by means of the BJH method from the N_2_ volume sorbed at the highest relative pressure.
- *Morphological characteristics:* (i) Metal contents were determined by ICP-OES using a Thermo Scientific iCAP 7400 duo instrument (Waltham, MA, USA). The samples were microwave digested in 5 mL HNO_3_ (70%, Fisher, Waltham, MA, USA) and 100 mg NH_4_F (≥98.0%, Sigma Aldrich, Darmstadt, Germany) at 190 °C (CEM–MARS microwave reactor, Matthews, NC, USA ) and finally diluted in 10% aqueous HNO_3_; (ii) Aberration-corrected High resolution TEM images were obtained on a JEOL 2100F (Tokyo, Japan) operated at 200 kV; (iii) A Bruker D8 Advance diffractometer (Karlsruhe, Germany) using monochromated Cu K_α1_ radiation (λ = 0.1542 nm) was used for Powder X-ray diffraction; (iv) The number of Ir surface sites and associated crystallite sizes were estimated via isothermal H_2_ chemisorption measurements, with a Quantachrome/ChemBet Pulsar TPR/TPD (Boynton Beach, Florida) chemisorption analyzer equipped with an Omnistar/Pfeiffer Vacuum (Aßlar, Germany) mass spectrometer as a detector. For this purpose, 100–300 mg of catalysts was loaded on the quartz U tube holder of the instrument and pretreated with a 1% H_2_/He mixture (15 mL/min) at 400 °C for 1 h, followed by flushing with N_2_ (15 mL/min) at 400 °C for 0.5 h, and cooling to room temperature under N_2_ flow. Then, pulses of pure hydrogen (280 μL) were injected until saturation and the total hydrogen uptake per gram of catalyst (chemisorbed H_2_, $V_{H2}^{chem.}$) was measured. These values were used to calculate the hydrogen to metal ratio, H/Ir, (i.e., the dispersion, *D_Ir_*) and the mean Ir crystallite size (${\bar{d}}_{Ir}$) using the following equations:
  (1) $$D_{Ir}(H/Ir)=\frac{V_{H2}^{chem.}\cdot F_{s}\cdot M_{Ir}}{V_{mol}\cdot X_{Ir}}$$
  (2) $${\bar{d}}_{Ir}\left(\mathrm{nm}\right)=\frac{6\cdot M_{Ir}\cdot 10^{20}}{D_{Ir}\cdot \rho_{Ir}\cdot \alpha_{Ir}\cdot N_{AV}}$$
  where, $V_{H2}^{chem.}$ is the H_2_-uptake in the chemisorption experiments (mL/g), *F_s_* is the hydrogen to metal correlation factor (= 2 assuming one-to-one correlation of adsorbed H atoms with metal sites, i.e., H-Ir), *M_Ir_* is the molecular weight of iridium (192.22 g/mol), *V_mol_* is the molar volume of an ideal gas at room temperature and 1 atm pressure (24,450 mL/mol), *X_Ir_* is the iridium content of the catalyst (*g_Ir_/g_cat_*), $\rho_{Ir}$ is the Ir metal density (22.5 g/mL), $\alpha_{Ir}$ is the area occupied by a surface Ir atom (0.12 nm^2^/atom), *N_AV_* = 6.023 × 10^23^ molecules/mol is the Avogadro number, and 10^20^ is a unit conversion factor when the units of parameters in Equation (2) are used as indicated herein.
- *Reducibility characteristics:* H_2_-TPR measurements were conducted to obtain the reducibility profiles and determine the total oxygen storage capacity (OSC) of both the supports and the corresponding catalysts, using the same TPR/TPD apparatus employed for H_2_ chemisorption measurements. Typically, an amount of ca. 150 mg of the material (support or catalyst) was loaded into the quartz U-tube holder of the instrument. Prior to data acquisition, samples were oxidized in situ at 750 °C for 30 min using a flow of 20% O_2_/He mixture followed by cooling to 25 °C in the same flow. The tube was then purged with He for 10 min. The TPR measurements were then conducted with a 15 mL/min flow of 1% H_2_/He and a heating ramp of 10 °C/min up to ~850 °C.

### 2.3. Catalytic Activity Measurements

Catalytic activity and stability testing was carried out in a continuous-flow single-pass tubular quartz (i.d. = 3 mm) fixed-bed reactor that could be operated under both differential (low conversion) and integral (high conversion) modes [17]. For each set of catalytic measurements, 50 mg of the catalyst (grain size 180–250 μm) was loaded into the reactor. The catalyst was held between two quartz wool plugs. The feed composition was regulated with MKS-247 mass flow controllers which were connected to the appropriate gas cylinders for the DRM reaction, i.e., CO_2_ (99.6%) and CH_4_ (99,995%), as well as 20% *v*/*v* O_2_/He and ultra-pure H_2_ for the case of in situ catalysts pretreatment. Τhe on-line system employed for the analysis of reactants and products consisted of a gas chromatograph (SHIMADZU GC-2014, Kyoto, Japan), equipped with a thermal conductivity detector, and Porapak N and Molecular Sieve 5A parallel columns) operated with Ar as carrier gas. The feed composition was 50 % *v*/*v* CH_4_ + 50% *v*/*v* CO_2_, simulating an equimolar biogas. For the differential reactor operation, the total feed flow rate (F_t_) was varied between ca. 100–200 mL/min (i.e., at weight-basis gas hourly space velocities, WGHSV = F_t_/w_cat_, between ca. 120,000–216,000 mL/g_cat_·h) in order to maintain CH_4_ and CO_2_ conversions below ~15%, thus ensuring intrinsic kinetic measurements. Integral operation was obtained by keeping the feed flow rate constant at 100 mL/min (WGHSV = 120,000 mL/g_cat_·h). The performance of the synthesized catalysts for the DRM reaction was studied in the temperature interval 500–750 °C.

To achieve a better comparison of the DRM activity of the catalysts, CH_4_ and CO_2_ consumption rates *r_i_* (*i:* CH_4_ or CO_2_) were expressed per Ir mass rather than per mass of catalyst, using Equation (3):

(3) $$r_{i\;}\left(\frac{\mathrm{mol}}{g_{Ir}\cdot s}\right)=\frac{F_{t}^{in}\cdot {\left[i\right]}^{in}-F_{t}^{out}\cdot {\left[i\right]}^{out}}{m_{cat}\cdot X_{Ir}}$$

where $F_{t}^{in}$ and $F_{t}^{out}$ are the total flow rates at the reactor inlet and outlet, respectively (in mol/s), m_cat_ is the mass of catalyst loaded in the reactor (0.050 g) and *X_Ir_* is the Ir content of the catalyst (g_Ir_/g_cat_).

The H_2_/CO molar ratio was calculated by

H_2_/CO = [H_2_]_out_/[CO]_out_ (4)

where [H_2_]^out^ and [CO]^out^ are the concentrations of H_2_ and CO respectively in the reactor effluent reformate gas.

The DRM stability after catalyst exposure to thermal sintering at oxidative conditions was evaluated from the deactivation of the catalysts, based on the normalized percentage reduction in CH_4_ and CO_2_ consumption rates as in Equation (5):

(5) $$\Delta r_{i}^{norm.}{}_{\;}\left(\%\right)=\frac{\left|r_{i}^{a}-r_{i}^{f}\right|}{r_{i}^{f}}\;\cdot 100\%$$

where *i* denotes CH_4_ or CO_2_ and the superscripts “*f*” and “*a*” refer to the *fresh* and thermally *aged* catalysts, respectively.

### 2.4. Carbon Deposition Measurements via TPO Experiments

Temperature programmed oxidation (TPO) was used to estimate the amount of the carbonaceous deposit present on the spent catalysts and to study their reactivity towards oxygen. In a typical measurement, 100 mg of catalyst, previously exposed to reaction conditions for 3 h, was placed in a quartz reactor and held in place by means of quartz wool. TPO was performed using a 10 °C/min linear heating ramp from room temperature to 750 °C under a constant flow (30 mL/min) of an oxidizing gas mixture (6.1% O_2_ in He). A K-type thermocouple was set to measure the temperature of the catalyst during the TPO experiment. Gas analysis of the reactor effluent was achieved by an on-line mass spectrometer (FL-9496 Balzers, Balzers Instruments, Liechtenstein) recording continuously the MS signals at m/z = 32 (O_2_), 28 (CO) and 44 (CO_2_). Calibration of the mass spectrometer was performed with the use of gas mixtures of known composition. 

It should be noted that the amount of CO produced in TPO experiments was negligible for all studied samples. Therefore, the TPO patterns reported here include only the CO_2_ response curves. The total amount of CO_2_ produced in each experiment was calculated by integrating the respective curve. Results were then used to determine the total amount of carbon deposits for each sample, expressed as μmol C/g_cat_.

## 3. Results and Discussion

### 3.1. Materials Characterization Results

**Figure 1:**
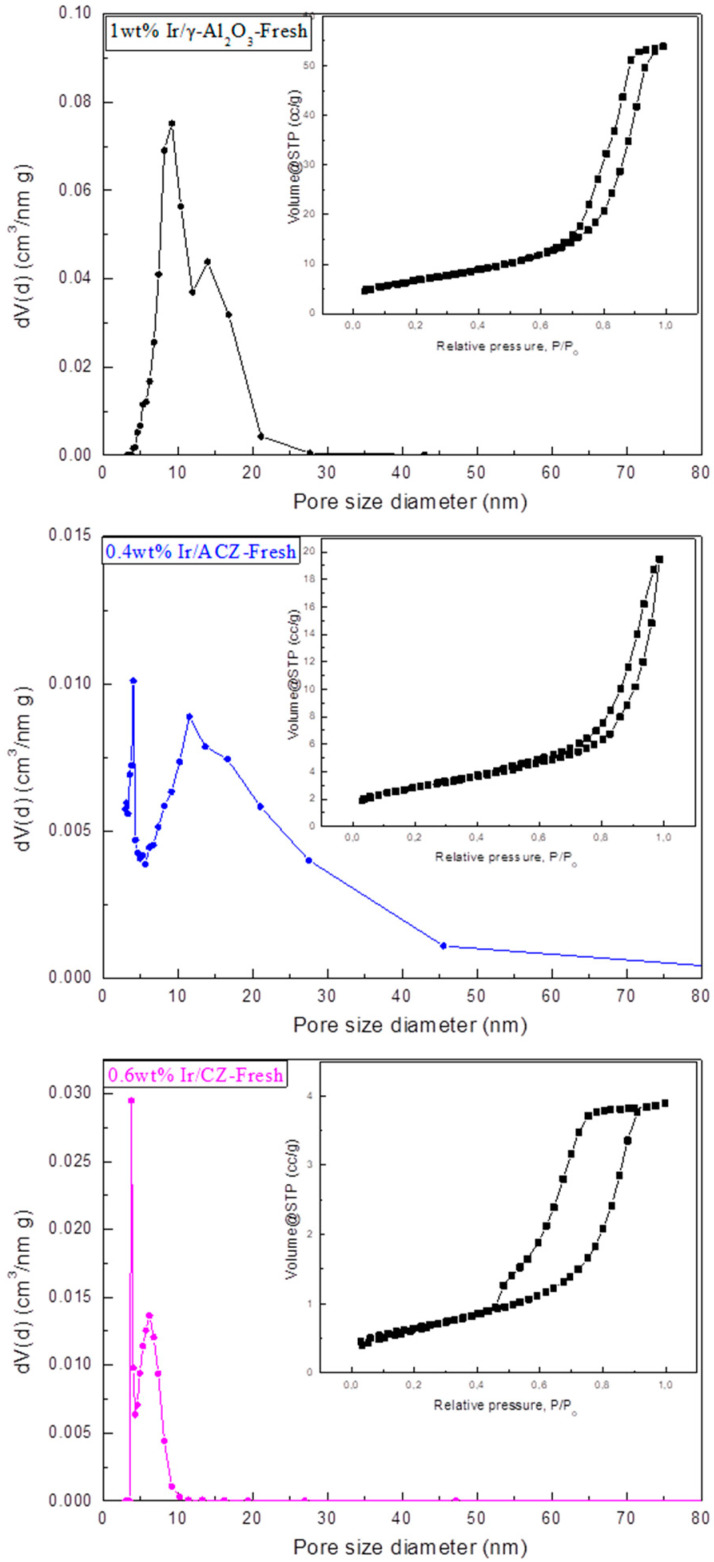
Pore size distribution and N_2_ adsorption–desorption isotherms (inset) of the as-prepared (fresh) Ir/γ-Al_2_O_3_, Ir/ACZ and Ir/CZ catalysts.

The pore size distributions and the corresponding N_2_ adsorption-desorption isotherms obtained at −196 °C for the three fresh catalysts are displayed in Figure 1. The textural characteristics of the catalysts and the corresponding unmetallized supports calculated from their N_2_ adsorption-desorption isotherms by means of the Brunauer-Emmett-Teller (BET) and Barrett–Joyner–Halenda (BJH) methods are summarized in Table 1. Both the total surface areas and the pore volumes of the fresh catalysts appear to be lower compared to the pristine supports indicating some pore blocking by the Ir nanoparticles. Based on the IUPAC classification, the isotherms observed in Figure 1 for the Ir/γ-Al_2_O_3_ and Ir/ACZ catalysts can be classified as type IV, similar to what is typically observed for mesoporous oxides, with an H1 hysteresis loop indicating the presence of a channel-like pore structure with spherical or cylindrical mesopores [79]. 

The absence of a distinct plateau on the isotherms at 0.8 < *P*/*P_o_* < 1 is most likely due to the presence of macroporosity and interparticle porosity. The absence of a steep rise at the beginning of the adsorption branch of the isotherm indicates the lack of micropores within the sample. Using BJH analysis, two or one pore size distributions of mesopores with diameters in the range of ca. 10–15 nm were obtained (Figure 1). Ir/CZ catalysts yielded type IV isotherms with H2-type hysteresis loop, which is usually associated with cylindrical-ink-bottle pores with principal distribution of ~7–8 nm, in agreement with findings in the literature [80].

**Figure 2:**
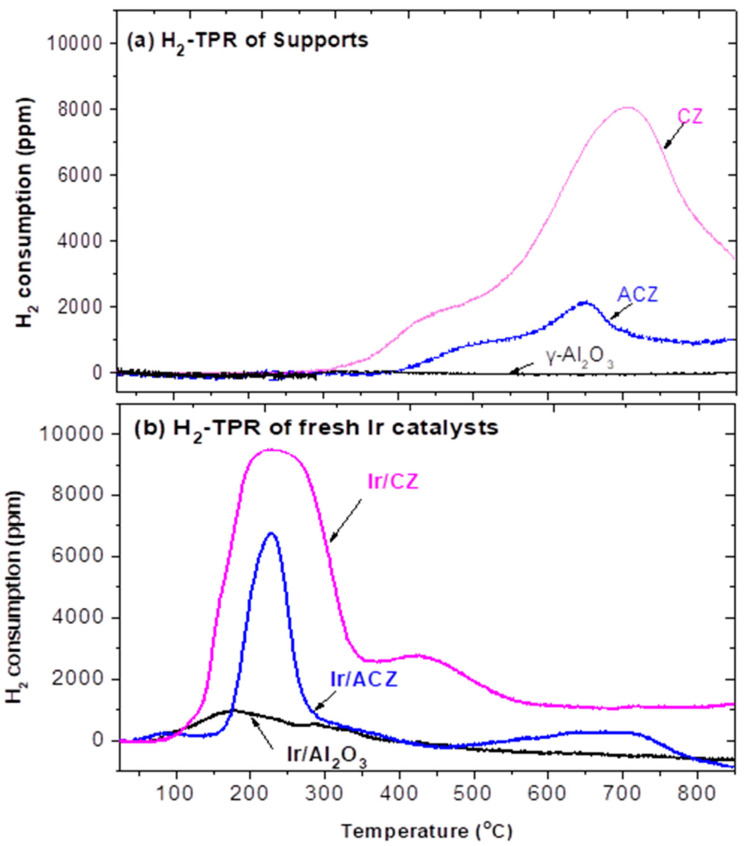
H_2_-TPR profiles of the γ-Al_2_O_3_, ACZ and CZ supports (**a**), and the counterpart Ir/γ-Al_2_O_3_, Ir/ACZ and Ir/CZ catalysts (**b**).

Figure 2 depicts the H_2_-TPR profiles for the γ-Al_2_O_3_, ACZ and CZ supports (Figure 2a) and for the counterpart Ir/γ-Al_2_O_3_, Ir/ACZ and Ir/CZ fresh catalysts (Figure 2b). The total amount of H_2_ consumed, determined from the integrated area of the respective TPR profiles in the time interval of the experiment, is used for the estimation of the total oxygen storage capacity (t-OSC) of the samples [68,75]. The resulting values are included in Table 1. Regarding the supports, their t-OSC apparently cover a wide range of values varying as γ-Al_2_O_3_ (0 μmol O_2_/g) < ACZ (110 μmol O_2_/g) < CZ (557 μmol O_2_/g), allowing a thorough study of the effect of OSC on the DRM activity and sintering characteristics of Ir nanoparticles deposited on them. The zero OSC value obtained for γ-Al_2_O_3_ reflects its nonreducible character, while the considerably high OSC values of ACZ and CZ reflect the Ce^4+^ → Ce^3+^ reduction of these CeO_2_-containing supports. The appearance of two relatively broad overlapping peaks located at ca. 450–500 and 650–700 °C (Figure 2a) is in accord with literature data for CZ-containing samples [81]. 

Dispersion of Ir crystallites on the supports caused an increase in the H_2_ consumption (Figure 2b) and the t-OSC of the Ir/γ-Al_2_O_3_, Ir/ACZ and Ir/CZ catalysts, which take values of 38, 176 and 601 μmol O_2_/g_cat_, respectively. The observed increase in the t-OSC values obtained for the metallized samples is close to that corresponding to the IrO_2_ → Ir^0^ reduction of the Ir content of the catalysts (52 μmol O_2_/g_cat_ for 1 wt% of Ir). Moreover, by comparing the H_2_-TPR profiles of supports and counterpart catalysts, it is apparent that the reducibility of the ceria was substantially favored in the presence of Ir since all hydrogen consumption peaks attributed to Ce^4+^ → Ce^3+^ reduction have been shifted toward considerably lower temperatures (ca. 100–450 °C; Figure 2b). This phenomenon is well known in the literature and has been attributed to the strong promotion of hydrogen spillover by the metal particles, in the absence of which the process is limited by H_2_ dissociation [81].

**Figure 3:**
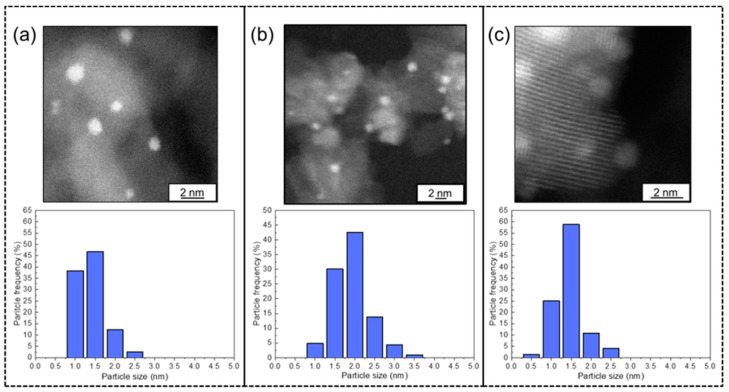
HR-TEM images and corresponding particle size distributions of the fresh (**a**) Ir/γ-Al_2_O_3_, (**b**) Ir/ACZ and (**c**) Ir/CZ catalysts.

The metal dispersions and corresponding nanoparticle sizes of the Ir/γ-Al_2_O_3_, Ir/ACZ and Ir/CZ catalysts estimated by means of the H_2_-chemisorption measurements are included in Table 1. As can be seen, the impregnation method employed for iridium dispersion on the supports achieved quite good dispersions (ca. 40–70%, Table 1) and small Ir crystallite sizes (ca. 1.0–1.7 nm) irrespective of the type and surface area of the support. The uniform distribution of Ir particles on the support surface and their small sizes were corroborated by HR-TEM measurements as shown in the representative HR-TEM images of Figure 3.

**Figure 4:**
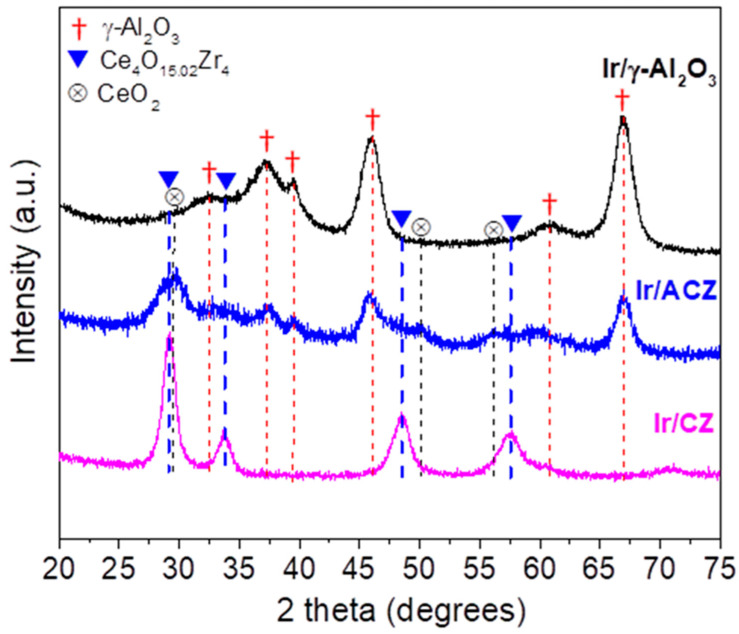
XRD diffractograms of fresh Ir/γ-Al_23_, Ir/ACZ and Ir/CZ catalysts.

Figure 4 shows representative PXRD data for the three fresh catalysts. Reflections corresponding to Ir particles, typically expected at 2θ = 40.7, 47.3 and 69.1 °C [74], were not detected due to the relatively small size of these nanoparticles in close agreement with the HR-TEM and H_2_ chemisorption results presented above; the diffractograms show reflections which can be only assigned to the oxide supports. Moreover, on Ir/CZ, the main crystal structure detected is that of a CZ solid solution, while CeO_2_ or ZrO_2_ distinct phases, if present, are at much lower contents to be detected. On Ir/ACZ, reflections corresponding mainly to γ-Al_2_O_3_ and a CZ solid solution [6] were detected, consistent with a mutual partial coating of these two phases at the nanometer scale for ACZ mixed oxides prepared by the co-precipitation method [77] similar to that employed herein.

### 3.2. Evaluation of Catalytic Performance and Stability

For performing, first, time-on-stream (TOS) stability measurements, 50 mg of catalyst were loaded in the reactor for 12 h at specific conditions (T = 750 °C; feed composition [CH_4_]^in^ = [CO_2_]^in^ = 50% at 1 bar; WGHSV = 120,000 mL/g·h). After these TOS experiments, comparative evaluation of catalytic performance of the synthesized materials was conducted by varying the temperature in the range of 500–750 °C, keeping the feed composition constant ([CH_4_]^in^ = [CO_2_]^in^ = 50% at a total pressure of 1 bar) with the reactor operated in the differential mode (i.e., varying gas space velocity if necessary so as to keep CH_4_ and CO_2_ conversions low). The results are presented below.

#### 3.2.1. Time-on-Stream Stability and Catalytic Performance during DRM

**Figure 5:**
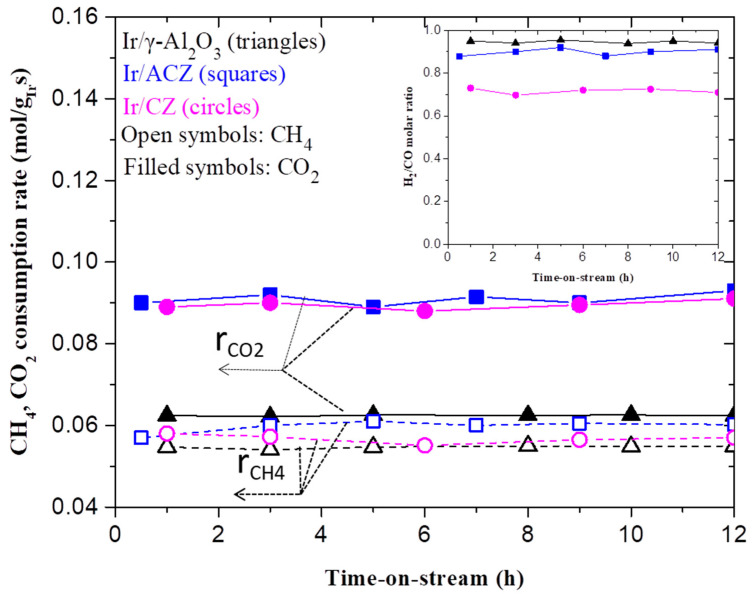
CH_4_ and CO_2_ consumption rates and the corresponding H_2_/CO molar ratios (inset) obtained with the fresh Ir/γ-Al_2_O_3_, Ir/ACZ and Ir/CZ catalysts as a function of time-on-stream at T = 750 °C and equimolar feed composition ([CH_4_]^in^ = [CO_2_]^in^ = 50% at a total pressure of 1 bar). Other conditions: WGHSV = 120,000 mL/g_cat_·h; catalyst mass w_cat_ = 50 mg. Open symbols and dashed lines indicate CH_4_ consumption rates; filled symbols and solid lines indicate CO_2_ consumption rates for Ir/γ-Al_2_O_3_ (triangles), Ir/ACZ (squares) and Ir/CZ (circles) catalysts.

The time-on-stream catalytic performance and stability results for the three catalysts is illustrated in Figure 5, which shows the time-dependent variation of the CO_2_ and CH_4_ rates of consumption, r_CO2_ and r_CH4,_ respectively (normalized per mass of active phase, i.e., mol/g_Ir_·s); the corresponding H_2_/CO molar ratios are depicted in the inset of Figure 5. It was found that all catalysts exhibited very good TOS stability. As will be discussed below, this good stability of Ir catalysts can be principally attributed to their low propensity for carbon deposition. Moreover, under the strongly reducing environment of DRM (i.e., CO + H_2_ reformate), it is expected that iridium would be present in its metal state, Ir^0^, which, according to the literature, is very stable and resistant to sintering [69]. Both factors contribute to the excellent TOS stability exhibited by Ir-based catalysts under DRM.

As can be seen in the inset of Figure 5, the H_2_/CO molar ratio of the catalysts was in all cases less than 1.0 and stable versus time-on-stream. At first glance, this shows that the rWGS reaction (R.4) also occurs under these conditions, resulting in a decrease in the H_2_/CO molar ratio to values lower than one corresponding to the stoichiometry of the DRM reaction (R.1) [23]. However, H_2_/CO molar ratio decreases in the order Ir/γ-Al_2_O_3_ > Ir/ACZ > Ir/CZ depending on the supporting material used. This reflects the increasing discrepancy between CO_2_ and CH_4_ consumption rates, the former varying in the opposite sense to the H_2_/CO molar ratios. The systematic increase in the relative CO_2_ conversion with increasing the OSC of the support (OSC_Al2O3_ = 0 μmolO_2_/g < OSC_ACZ_ = 110 μmolO_2_/g < OSC_CZ_ = 557 μmolO_2_/g), and the concomitant increase in CO formation relative to H_2,_ indicates the occurrence of support-induced modifications of the Ir surface chemistry. This issue is discussed below in connection with the intrinsic activity data.

#### 3.2.2. DRM Performance under Differential Conditions (Intrinsic Activity)

**Figure 6:**
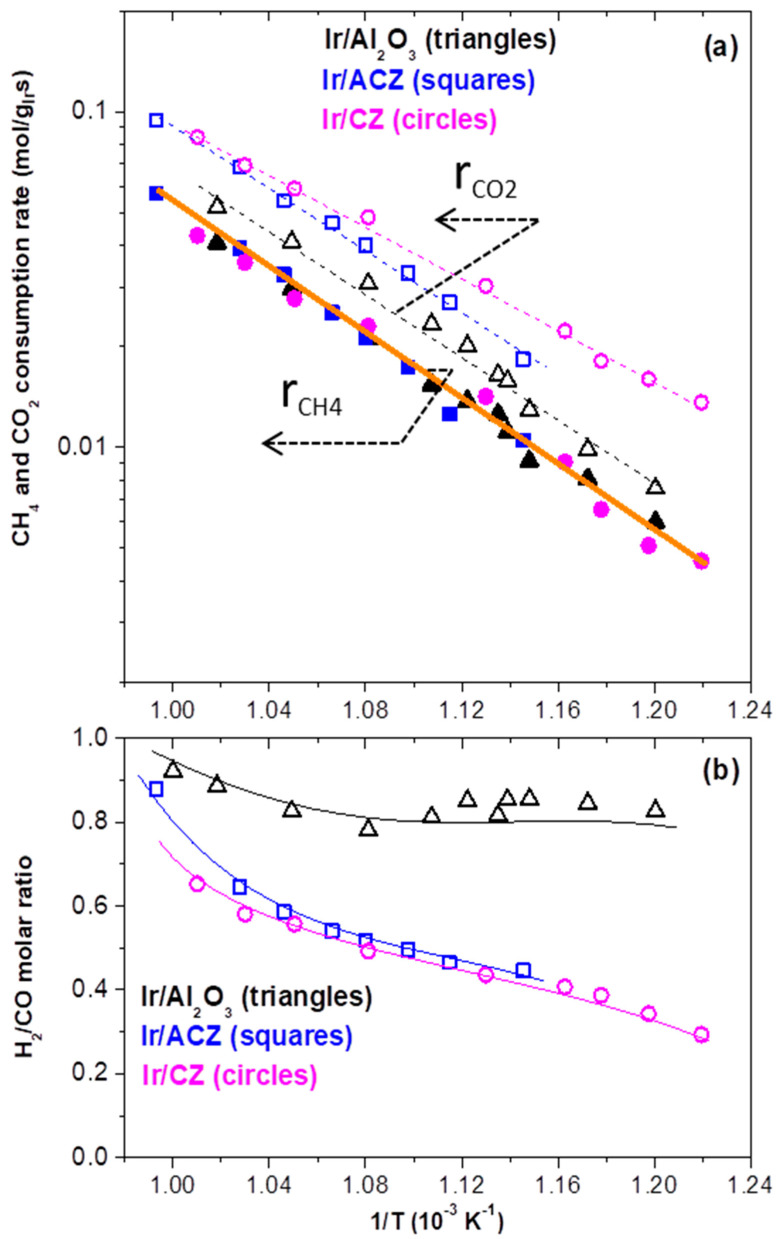
Arrhenius plots showing the temperature dependence of (**a**) the CH_4_ (closed symbols) and CO_2_ (open symbols) consumption rates, and (**b**) the corresponding H_2_/CO molar ratios, obtained for fresh Ir/Al_2_O_3_, Ir/ACZ and Ir/CZ catalysts. Experimental conditions: equimolar feed composition ([CH_4_] = [CO_2_] = 50 % at 1 bar); catalyst mass w_cat_ = 50 mg; WGHSV = 120,000–200,000 mL/g_cat_·h.

Results of intrinsic activity measurements acquired with Ir/Al_2_O_3_, Ir/ACZ and Ir/CZ catalysts under differential reactor operation, i.e., at low CH_4_ and CO_2_ conversions (ca. 5–15%) in order to eliminate mass and/or thermal transport constrains, are shown in the Arrhenius plots of Figure 6. Similar to results presented in Figure 4, methane and carbon dioxide consumption rates are normalized per mass of the active phase (Ir) contained in the catalyst loaded in the reactor, i.e., as moles of consumed reactant per mass of Ir per second, in order to take into account the different metal loading of the 1 wt% Ir/Al_2_O_3_, 0.4 wt% Ir/ACZ and 0.6 wt% Ir/CZ catalysts. Therefore, variations in the catalytic rates mainly reflect possible metal-support interactions [82] originating from the large variation in oxygen storage capacity and lability between the three supports used (Table 1). It is apparent from Figure 6a that, while all three catalysts exhibit practically the same methane consumption rates, the CO_2_ consumption rates vary appreciably depending on the nature of the support. In particular, iridium supported on CZ and ACZ exhibits a much higher intrinsic activity compared to Ir/γ-Al_2_O_3_, with Ir/CZ being overall the most active, especially at lower temperatures. In addition, the CO_2_ consumption apparent activation energies (*E_a_*) are lower for the CZ-containing catalysts with *E_a_* decreasing from 91.3 kJ/mol on Ir/γ-Al_2_O_3_ to 88.5 kJ/mol on Ir/ACZ and to 73.5 kJ/mol on Ir/CZ (Table 2). Regarding the CH_4_ consumption rate, all catalysts exhibited similar E_a_ values of about 92.4 kJ/mol (Figure 6a). This behavior suggests that a bifunctional reaction mechanism takes place, which is more pronounced on supports with higher oxygen storage capacity (labile lattice oxygen). Specifically, surface oxygen vacancies, which are abundant on the ACZ− and CZ-supported catalysts, could activate CO_2_ by adsorption and disproportionation to CO + O. Subsequently regenerated labile oxygen ions could then promote the oxidation of carbon deposited on the support and simultaneously increase the oxygen ion spillover onto the Ir particles, creating an [O^δ−^,δ^+^] effective double layer on their surface. This results in alteration of the electronic state, properties and surface chemistry of Ir nanoparticles by metal-support interactions. The effect of the as-created double layer on the electronic state of noble metal particles has been recently revealed by state-of-the-art XPS measurements on Rh nanoparticle catalysts dispersed on γ-Al_2_O_3__−_ and CZ-containing supports similar to those used in the present work [6]. It was shown that, under DRM conditions, the Rh species existed mainly in the metallic state (Rh^0^) and the relative Rh^0^ content decreased with decreasing the OSC of the support, following the order Rh/CZ (Rh^0^ = 100%) > Rh/ACZ (Rh^0^ = 72%) > Rh/γ-Al_2_O_3_ (Rh^0^ = 55%). It is reasonable to suggest that a qualitatively similar effect of the support’s OSC on the electronic state of the dispersed metal is also operable in the case of Ir nanoparticles, thereby favoring their DRM activity when dispersed on CZ-containing supports compared to γ-Al_2_O_3_. In addition, the spillover oxygen species can also act as promoters of the DRM reaction before they react with CH_4_-derived adsorbed carbonaceous species on Ir sites [83]. The CO-enriched syngas produced by Ir/ACZ and Ir/CZ catalysts (Figure 6b) is consistent with this interpretation as well as with temperature programmed oxidation (TPO) results conducted over used catalysts described below. The enhanced basicity of ceria-containing supports, which has been proposed to promote the reverse water gas shift reaction [1,2,6,7], may also explain the higher CO content and the lower H_2_/CO molar ratio of the reformate, observed for the CZ-containing catalysts.

#### 3.2.3. Carbon Deposition Measurements

**Figure 7:**
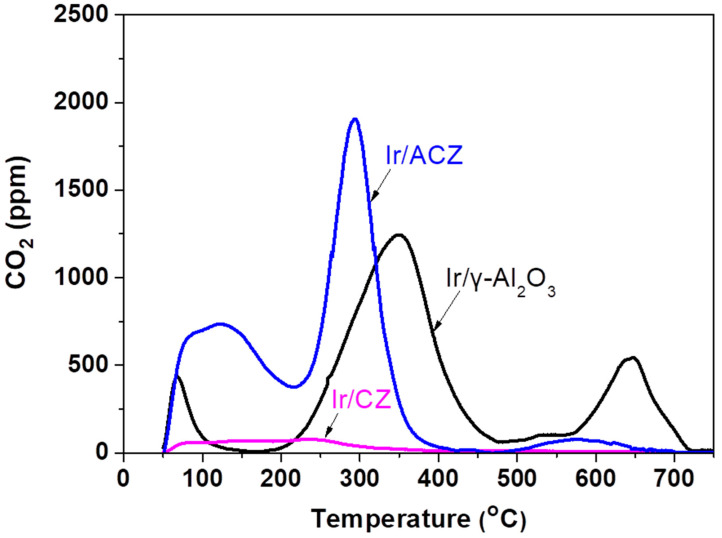
Temperature programmed oxidation profiles (CO_2_ production versus temperature) of fresh Ir/γ-Al_2_O_3_, Ir/ACZ and Ir/CZ catalysts after exposure to DRM reaction conditions (i.e., T = 750 °C, [CO_2_] = [CH_4_] = 50% at 1 bar, m_cat_ = 100 mg, WGHSV = 60,000 mL/g_cat_·h). Temperature ramp of TPO was 10 °C/min from room temperature to 750 °C under a stream of 6.1% O_2_ in He.

The total amount and types of carbon species deposited on the three catalysts after exposing them to DRM reaction conditions for 3 h were evaluated by TPO experiments. The obtained results are shown in Figure 7 as temperature profiles of CO_2_ produced since CO production was found to be negligible in all cases. Table 3 shows the quantification of the data presented in Figure 7 in terms of the mean rate of carbon deposition and the total amount of carbon deposited during 3 h of DRM operation (using the time interval value of the total area of all peaks appearing in each TPO profile). The extent of carbon deposition appears to be relatively low in all three cases, in agreement with the good time-on-stream stability of the catalysts shown in Figure 5 and in accordance with the well-known excellent resistance of Ir catalysts to coking under DRM [2,49]. However, significant differences in the amount and type of carbon deposited on the catalysts studied are apparent in Figure 7. Specifically, the total amount of carbon deposits followed the order Ir/γ-Al_2_O_3_ ~ Ir/ACZ >> Ir/CZ (Figure 7 and Table 3). Thus, the use of CZ with its high OSC value as support for Ir nanoparticles results in a catalyst with very low propensity toward carbon accumulation during DRM, which is similar to and indeed better than that of Rh/CZ catalysts [6].

**Scheme 1:**
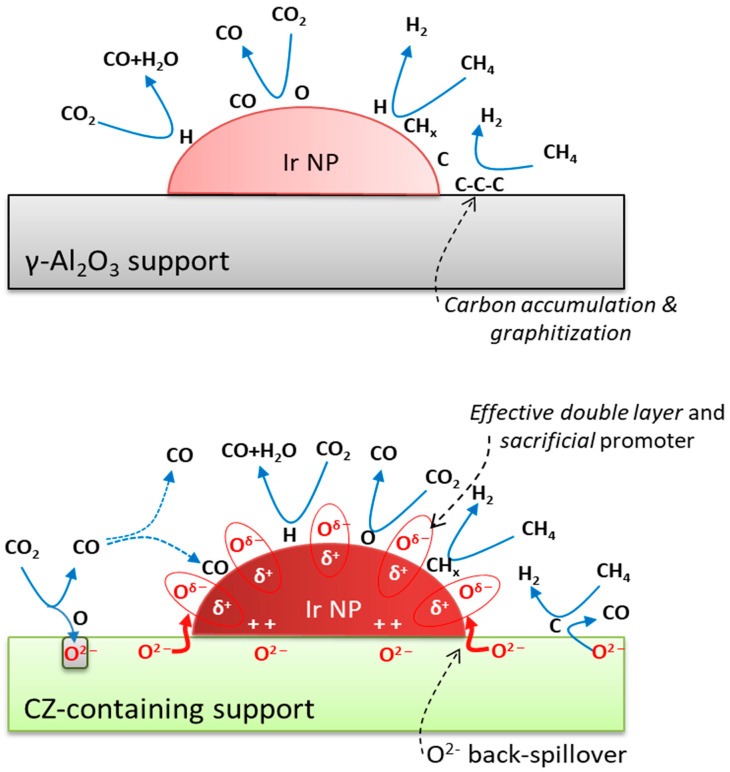
Graphical representation of the DRM reaction pathways over Ir nanoparticles (Ir NP) dispersed on γ-Al_2_O_3_ and CZ-containing supports. The promotional effects induced by CZ-containing supports with high OSC values originate from the presence of the surface oxygen vacancies, labile O^2−^ species and back-spillover phenomena.

Apart from this, the support strongly influences the type of the carbon species formed. The TPO profile of Ir/γ-Al_2_O_3_ shows three principal peaks at ca. 70, 360 and 650 °C attributed to reactive superficial carbide species, readily oxidized amorphous carbonaceous species and hard to oxidize graphitic carbon allotropes, respectively [1,6,20,48,56,84,85]. The amorphous carbonaceous species (TPO peak at 360 °C) are formed via dehydrogenation of adsorbed hydrocarbon fragments and are generally reactive under reforming reaction conditions [86]. However, if these species are not removed from the catalyst surface at sufficiently high rates, as in the case of the Ir/γ-Al_2_O_3_ catalyst, they can be converted to polymeric carbon and, eventually, to graphitic carbon species, which are much less reactive (TPO peak at 650 °C). Graphitic carbon deposits are substantially suppressed on Ir/ACZ catalysts and practically absent on Ir/CZ. This indicates that the high oxygen ion capacity and mobility on the CZ support enhances the gasification rate of amorphous carbon, which becomes higher than the carbon deposition rate, thereby resulting in low or negligible amounts of accumulated carbon, especially graphitic, on the catalyst surface [56]. A bifunctional reaction mechanism is at work where labile oxygen species from the reducible Ce-containing support react with carbon deposits in the vicinity of Ir crystallites and clean the surface. The oxygen vacancies of the support are replenished by oxygen atoms originating from the dissociative adsorption of CO_2_. Scheme 1 summarizes the bifunctional reaction mechanism according to our experimental findings.

#### 3.2.4. Evaluation of DRM Catalyst Stability after Exposure to Oxidative Thermal Aging

In order to evaluate their oxidative thermal aging stability, the Ir/γ-Al_2_O_3_, Ir/ACZ and Ir/CZ catalysts were tested at fixed DRM conditions (T = 750 °C, [CH_4_] = [CO_2_] = 50% *v*/*v* at 1 bar, WGHSV = 120,000 mL/g_cat_·h) on samples which had been in situ exposed to two consecutive aging protocols, i.e., “aged@650” and “aged@750” as described in the experimental section. It is worth emphasizing that BET-BJH analysis of the N_2_ physical adsorption-desorption isotherms conducted on the thermally aged catalysts showed rather small changes in their textural characteristics even after the second aging protocol (aged@750). On the other hand, regarding Ir particle sizes, the aging procedure resulted in significant Ir particle growth on Ir/γ-Al_2_O_3_ (from 1.0 on fresh to 13.6 nm on aged@750) but only marginal on Ir/ACZ (from 1.7–2.1 nm) and Ir/CZ (from 1.3–1.6 nm) catalysts. The characterization results of aged@750 catalysts are summarized in Table 4.

**Figure 8:**
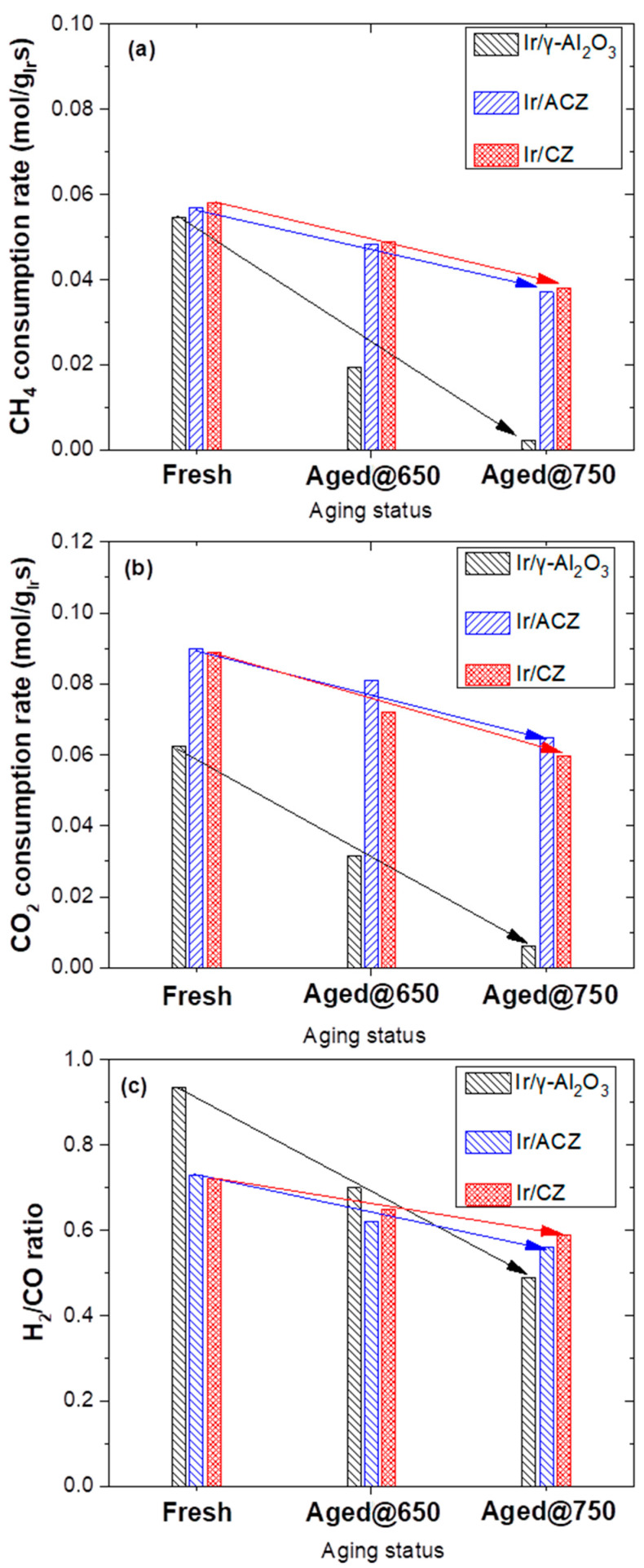
The effects of consecutive oxidative thermal aging on the DRM performance, i.e. methane consumption rate (**a**), carbon dioxide consumption rate (**b**) and H_2_/CO molar ratio (**c**), of Ir/γ-Al_2_O_3_, Ir/ACZ and Ir/CZ catalysts. Aging protocols: (i) Aged@650 (2 h in situ oxidation with 50 mL/min flow of 20% O_2_/He at 650 °C); (ii) Aged@750 (consecutive 2 h additional in situ oxidation with 50 mL/min flow of 20% O_2_/He at 750 °C). DRM performance was evaluated at 750 °C, equimolar feed composition [CH_4_] = [CO_2_] = 50% at a total pressure of 1 bar, m_cat_ = 50 mg, WGHSV = 120,000 mL/g_cat_·h. DRM performance values given are the mean values of ca. 5–6 measurements taken in a period of ~ 6 h of stable time-on-stream operation.

The comparative catalytic evaluation of the fresh and aged catalysts under DRM conditions is depicted in Figure 8 and shows the following features: Ir/γ-Al_2_O_3_ catalyst demonstrated a significant deactivation in both CH_4_ and CO_2_ consumption rates (Figure 8a,b and Table 5), which led to almost full loss of its activity (>90%, Table 5) after the second aging at 750 °C. In contrast, both Ir/ACZ and Ir/CZ underwent a slight deactivation (ca. 15%) after the first aging and some more (ca. 32%) after the second aging, thus substantially retaining their initial DRM activity (Table 5) despite the fact that Ir particles are strongly prone to agglomeration under such high temperature oxidative thermal sintering conditions [66,69]. Obviously, the existence of CeO_2_ in the support endows the Ir/ACZ and Ir/CZ catalysts with a substantial anti-sintering characteristic of Ir particles (Table 4), in agreement with results reported in our previous studies [68,74,75].

The sintering-resistance mechanism induced by CeO_2_-containing supports has been thoroughly elucidated in refs. [68,74,75] in terms of metal-support interactions and the presence of a high concentration of oxygen vacancies on the surface of the support. Briefly, the model of anti-sintering behavior involves two determining factors: (i) an O^δ−^ layer spontaneously forming on the Ir particle’s surface, due to thermally-driven oxygen back-spillover from high oxygen ion lability supports; the presence of this layer minimizes particle migration and coalescence (PMC) of large iridium particles via the resulting inter-particle electrostatic repulsion, and (ii) an entrapment of Ir atoms which have been detached from large Ir crystallites by surface oxygen vacancies in the support, thus suppressing surface diffusion of such species and their subsequent attachment to larger particles (Ostwald ripening mechanism) [68,74,75].

## 4. Conclusions

The dry reforming of methane to syngas production was investigated over Ir catalysts dispersed on three oxide supports (γ-Al_2_O_3_, ACZ and CZ) characterized by different oxygen storage capacity (OSC). The aim was to elucidate the effects of this important supporting material’s property on the catalyst’s intrinsic activity, selectivity, time-on-stream stability, and resistance to sintering and carbon deposition. Results obtained can be summarized as follows:

1. The use of ACZ and CZ supports with sufficient OSC and surface oxygen vacancies substantially enhances the CO_2_ consumption rate but does not affect appreciably the CH_4_ consumption rate. This implies the occurrence of a bifunctional reaction mechanism according to which the oxygen vacancies and labile oxygen ions of the support promote dissociative CO_2_ adsorption and the production of a CO-enriched syngas. Such synergistic phenomena are absent on the γ-Al_2_O_3_-supported catalyst, which is characterized by negligible OSC and surface oxygen vacancies and, concomitantly, lower CO_2_ consumption rates. In addition, the apparent activation energy for CO_2_ consumption decreases with increasing OSC value of the support, thus favoring the DRM reaction, especially in the low-temperature region.
2. All catalysts exhibit excellent time-on-stream stability regardless of the OSC of the support. This is attributed to the intrinsically low propensity of Ir for the formation and accumulation of carbon deposits and the predominance of the thermally stable metallic Ir phase under highly reducing DRM reaction conditions (CO+H_2_ reformate), which prevents particle agglomeration.
3. The support OSC strongly affects the amount and type of carbon deposits accumulated on the catalyst surface following exposure to reaction conditions. The formation of graphitic carbon is significantly suppressed over Ir/ACZ, compared to Ir/γ-Al_2_O_3_, and is negligible for the Ir/CZ sample. Interestingly, the latter catalyst does not promote the accumulation of any type of carbon deposits during DRM, verifying the important role of labile O^2−^ species of the support on the gasification rate of surface carbon species.
4. Oxidative thermal aging experiments demonstrated that the OSC of the support is a key factor in preventing iridium particle growth (sintering) despite the fact that IrO_2_ is highly prone to agglomeration under such conditions. Thus, Ir/ACZ and Ir/CZ (but not Ir/γ-Al_2_O_3_) maintain their initial DRM activity, even after severe thermal aging. The spontaneous, thermally driven O^2−^ back-spillover from the high oxygen ion lability supports to the Ir particle’s surface is responsible for this anti-sintering behavior.
5. These advantageous features of iridium supported on high-oxygen storage capacity and lability supports indicate that such catalysts can be cost effective (low Ir-loading), stable (regardless of the oxidizing or reducing environments) and highly active, especially for the low-temperature DRM process, which remains a challenging and attractive industrial application.

## Figures and Tables

**Table 1:** Textural, morphological and reducibility characteristics of the γ-Al_2_O_3_, ACZ and CZ supports and the corresponding as-prepared (fresh) Ir/γ-Al_2_O_3_, Ir/ACZ and Ir/CZ catalysts.

Supports and Catalysts	Ir-Content (wt.%) ^a^	S_BET_ (cm^2^/g)	Total Pore Volume (cm^3^/g)	OSC (μmol O^2^/g)	$V_{H2}^{chem.}({\mathrm{cm}}^{3}/g)$	D_Ir_ (H/Ir)	Mean Ir Particle Size (nm) ^b^ H_2_-chem/TEM
γ-Al_2_O_3_	−	178	0.60	0	−	−	−
Ir/γ-Al_2_O_3_	1.0	167	0.57	38	0.45	0.70	1.0/1.2 ± 0.3
ACZ: 80 wt% Al_2_O_3_−20 wt% Ce_0.5_Zr_0.5_O_2__−__δ_	−	149	0.29	110	−	−	−
Ir/ACZ	0.4	73	0.22	176	0.103	0.41	1.7/1.8 ± 0.5
CZ: Ce_0.5_Zr_0.5_O_2__−__δ_	−	22	0.05	557	−	−	−
Ir/CZ	0.6	17	0.04	601	0.231	0.61	1.2/1.3 ± 0.4

^a^ Ir content was measured by means of ICP-OES. ^b^ The mean metal particle size compares values obtained by isothermal H_2_-chemisorption experiments and HRTEM images.

**Table 2:** Support-induced effects on apparent activation energies and pre-exponential factors of CO_2_ consumption rate during DRM over Ir/γ-Al_2_O_3_, Ir/ACZ and Ir/CZ catalysts.

Catalyst	Support *t-OSC* (μmol O_2_/g)	*r_CO2_* Apparent Activation Energy (*E_a_*) (kJ/mol)	Pre-Exponential Factor ( $r_{CO2}^{o}$) (mol CO_2_/ g_Ir_·s)
Ir/γ-Al_2_O_3_	0	91.3 ± 0.4	4146.4 ± 1.7
Ir/ACZ	110	88.5 ± 0.3	3827.6 ± 1.3
Ir/CZ	557	73.5 ± 0.2	645.5 ± 1.2

**Table 3:** Amount of carbon deposited and deposition rate after 3 h of DRM operation.

Catalyst	Total Amount of Carbon Deposited (μmol C/g_cat_)	Mean Carbon Deposition Rate (μmol C/g_cat_·h)
Ir/γ-Al_2_O_3_	324	108
Ir/ACZ	328	109
Ir/CZ	27	9

**Table 4:** Textural and morphological characteristics of the “*aged@750*” catalysts.

Catalyst	S_BET_ (m^2^/g)	Total Pore Volume (cm^3^/g)	$V_{H2}^{chem.}$ (cm^3^/g)	D_Ir_ (H/Ir)	Mean Ir Particle Size (nm) ^a^
Ir/γ-Al_2_O_3_-Aged@750	140	0.31	0.033	0.05	13.6
Ir/ACZ-Aged@750	64	0.20	0.087	0.34	2.1
Ir/CZ-Aged@750	16	0.04	0.169	0.44	1.6

**^a^** The mean Ir particle sizes obtained by isothermal H_2_-chemisorption experiments.

**Table 5:** Normalized Percentage deactivation of catalysts DRM performance, $\Delta r_{CH_{4}}^{norm.}{}_{\;}\left(\%\right)\;$ and $\Delta r_{CH_{4}}^{norm.}{}_{\;}\left(\%\right)$ (Equation (5)), after exposure of the catalysts to oxidative thermal aging protocols.

Catalyst	Support *t-OSC* (μmol O_2_/g_supp_)	$\Delta r_{CH_{4}}^{norm.}$ (%) 1st Aging 2nd Aging		$\Delta r_{CO_{2}}^{norm.}$ (%) 1st Aging 2nd Aging		**Ref.**
Ir/γ-Al_2_O_3_	0	64.5 ^a^	95.7 ^b^	49.5 ^a^	90.2 ^b^	This work
Ir/ACZ	110	15.1 ^a^	35.1 ^b^	10.0 ^a^	27.8 ^b^	This work
Ir/CZ	557	15.5 ^a^	34.5 ^b^	19.1 ^a^	33.0 ^b^	This work
Ir/γ-Al_2_O_3_	0	35.6 ^c^	68.8 ^d^	29.0 ^c^	58.1 ^d^	[50]
Ir/YSZ	5.7	28.3 ^c^	81.5 ^d^	14.7 ^c^	69.0 ^d^	[50]
Ir/GDC	186	5.9 ^c^	5.2 ^d^	0.0 ^c^	0.0 ^d^	[50]

^a^ Aging protocol: 2 h at 650 °C (Aged@650); DRM testing conditions: [CH_4_] = 50%, [CO_2_] = 50% (CO_2_/CH_4_ = 1/1), WGHSV = 120,000 mL/g_cat_·h. ^b^ Aging protocol: 2 h at 650 °C plus 2 h at 750 °C (Aged@750); DRM testing conditions: [CH_4_] = 50%, [CO_2_] = 50% (CO_2_/CH_4_ = /1), WGHSV = 120,000 mL/g_cat_·h. ^c^ Aging protocol: 1 h at 750 °C; DRM testing conditions: [CH_4_] = 35.5%, [CO_2_] = 64.5% (CO_2_/CH_4_ = 1.8/1), WGHSV = 30,000 mL/g_cat_·h. ^d^ Aging protocol: 2 h at 750 °C; DRM testing conditions: [CH_4_] = 35.5%, [CO_2_] = 64.5% (CO_2_/CH_4_ = 1.8/1), WGHSV = 30,000 mL/g_cat_·h.
